# Mass Depopulation of Swine during COVID-19: An Exploration of Swine Veterinarians’ Perspectives

**DOI:** 10.3390/vetsci9100563

**Published:** 2022-10-13

**Authors:** Cori Bussolari, Wendy Packman, Jennifer Currin-McCulloch, Elizabeth Strand, Lori Kogan

**Affiliations:** 1 Department of Counseling Psychology, University of San Francisco, San Francisco, CA 94117, USA; 2 Department of Psychology, Palo Alto University, Palo Alto, CA 94304, USA; 3 School of Social Work, Colorado State University, Fort Collins, CO 80523, USA; 4 Department of Veterinary Medicine, University of Tennessee, Knoxville, TN 37996, USA; 5 Department of Clinical Sciences, Colorado State University, Fort Collins, CO 80523, USA

**Keywords:** depopulation, large animal, swine, veterinarian, COVID-19, well-being

## Abstract

**Simple Summary:**

This study was designed to better understand the mental health of U.S. swine veterinarians who were involved in the mass depopulation events related to COVID-19. A total of 25 swine veterinarians, participants from a larger quantitative study, volunteered to be interviewed about their experiences related to the COVID-19 mass depopulation event. The themes that arose from these interviews included: (1) the need to be better prepared for crisis events; (2) lack of public understanding; (3) moral distress; (4) empathy for others, especially young veterinarians; (5) sources of support; (6) pride, honor and gratitude; and (7) an overarching theme of emotional distancing and detachment—concerns external to one’s own mental health. Based on these results, we recommend additional training and supportive services for those who might be involved in future depopulation efforts.

**Abstract:**

This qualitative study (n = 25) was created to better understand the mental health of U.S. swine veterinarians who were involved in the mass depopulation events related to COVID-19. A total of 25 swine veterinarians, participants in a previous larger quantitative study, volunteered to be interviewed about their experiences related to the COVID-19 mass depopulation event. Themes that emerged from these interviews included: (1) the need to be better prepared for crisis events; (2) lack of public understanding; (3) moral distress; (4) empathy for others, especially young veterinarians; (5) sources of support; (6) pride, honor and gratitude; and (7) an overarching theme of emotional distancing and detachment—concerns external to one’s own mental health. Based on our results, we recommend additional training and supportive services for those who might be involved in future depopulation efforts. Additionally, we suggest that the veterinary profession prioritize public education campaigns to help the public better understand the need for depopulation.

## 1. Introduction

Euthanasia, the act of taking life to eliminate suffering, is an established part of veterinary practice and provides an invaluable tool by which veterinarians can bring relief to both animals and those who care for them [1,2]. Numerous studies, however, have suggested that conducting euthanasia can have negative psychological effects on veterinary professionals including burnout, compassion fatigue, somatic problems, and diminished job satisfaction [3,4,5,6,7]. Research has shown that veterinarians and others involved in killing animals have increased risks of mental health problems [8,9,10,11] and unfortunately, understanding the rationale behind the decision to kill is often not sufficient to mitigate these risks [8,11,12]. Many of those who euthanize animals suffer from perpetration-induced traumatic stress (PITS), a form of post-traumatic stress disorder (PTSD) [13,14]. Instead of involving a direct threat to the individual like traditional PTSD, PITS occurs when the threat is to one’s ethical character; in this case, veterinarians who care for animals, and at the same time, must end their lives [8,15,16].

As a result, both euthanasia and depopulation can lead to moral distress [17,18,19], often caused by the “caring–killing paradox” [5]; the moral challenge of needing to take the life of an animal, while simultaneously feeling compassion toward animals [5,17,20]. Holding these conflicting sentiments can be onerous. This is compounded by the fact that, by nature, veterinary medicine includes professional obligations to multiple entities including the animal, the owner, those in the veterinary profession, and society [21]. Moral distress may arise when these obligations clash with each other and/or a veterinarians’ own morals [21].

The moral challenges for those engaged in depopulation efforts share similarities with those who perform euthanasia, but also include unique challenges [11,22,23]. Depopulation is the killing of animals, with as much consideration as possible given to animal welfare, in response to emergency circumstances (e.g., disease control, natural or human-made disasters, etc.) [24]. The challenge facing veterinarians and others tasked with depopulation is the need for an aggressive, rapid response to an emergency situation while balancing animal and human welfare concerns. Even though human health and safety is often the priority in depopulation events, all those involved are encouraged to take the necessary steps to minimize animal suffering [23]. Veterinarians often oversee and lead emergency depopulation processes, providing guidance and oversight related to animal welfare while weighing the immediate risk to humans, other animals, and the environment [11,23,24].

The beginning months of the COVID-19 pandemic led to the need for a mass swine depopulation event within the United States. As packing plants began diagnosing COVID-19 in their workers in March 2020, the movement of swine to slaughter by U.S. swine producers was quickly affected [25]. The temporary closure of slaughter and meat processing plants necessitated the immediate need for depopulation, sometimes referred to as "Welfare Slaughter", a term used to describe the killing of noninfected, healthy animals in response to a foreign animal disease outbreak [8,26,27,28]. As explained by the American Association of Swine Veterinarians (AASV), depopulation may be necessary for several reasons, including a disruption in the production process that can negatively impact animal welfare [29,30,31]. Numerous methods are employed for depopulation including bullets, captive bolts, electrical methods, gas mixtures, and lethal injections [32]. Several factors are considered when determining the best depopulation method, including the nature of the disease, the number of animals to be killed, and the resources required. Animal welfare is paramount during depopulation efforts, and the veterinary ethics of care requires that the method chosen is one that attempts to minimize animal suffering. Carbon dioxide is typically the depopulation method used for large numbers of animals, yet the COVID-19 pandemic created a unique set of circumstances that made carbon dioxide unavailable in many parts of the United States [33]. Because many ethanol plants were not operating due to COVID-19 related shutdowns (carbon dioxide is a byproduct of ethanol), and other methods were explored and not found feasible, some farms utilized VSD+ (ventilation shutdown with the addition of high temperature, a high concentration of carbon dioxide, or both) or VSD+TH (ventilation shutdown with the addition of high temperature and humidity) [33]. These methods have been classified by the American Veterinary Medical Association (AVMA) as permitted in constrained circumstances [24]. Many swine farms during the initial months of the COVID-19 pandemic were faced with a lack of options for more preferred, traditional methods of depopulation. Many swine veterinarians at this time were part of the teams that determined the necessity to depopulate and as a result, led COVID-19 emergency mass swine depopulation processes.

Concern about the potential psychosocial impact of this depopulation event on swine veterinarians’ mental health led Baysinger and Kogan [11] to conduct a quantitative study with swine veterinarians in the United States. Their study found swine veterinarians involved in the depopulation event reported higher levels of burnout compared to those not directly involved [11]. They also found that even though more than 50% of the swine veterinarians surveyed recognized the importance of mental health services, only a minority reported actually receiving these services [11]. This qualitative study was designed to better understand the experiences of US swine veterinarians during this challenging time by interviewing a subsample of participants from the Baysinger and Kogan [11] study.

## 2. Materials and Methods

### 2.1. Study Sample

Baysinger and Kogan’s study [11] employed a web-based survey to assess swine veterinarians’ experiences and feelings regarding the COVID-19 depopulation event. The survey was distributed between December 2020–January 2021 via an email through the American Association of Swine Veterinarians (AASV). At the end of the survey, participants were asked if they would like to volunteer to participate in a follow-up interview. Those who indicated they were interested were directed to a new website in which they were asked to provide their name and contact information. In this way, all surveys remained anonymous. All participants interviewed were offered a $50 Amazon gift card for their time. A total of 25 swine veterinarians agreed to be interviewed; 17 who were involved in the depopulation effort and 8 who identified as “non-involved”. This research was approved by Colorado State University’s Institutional Review Board (#2118).

### 2.2. Data Collection

Participants completed an individual semi-structured interview lasting from 30 to 90 minutes by video conference platform or telephone. The interview centered on the events surrounding the swine depopulation mandate during the first months of COVID-19 and included questions about their level of perceived support and their mental health needs. Each interview was recorded, transcribed, and reviewed by the research team to verify accuracy of data. The transcribed Microsoft Word documents were uploaded into a Microsoft Excel format to organize the data to support a systematic approach needed to code and analyze the qualitative data.

### 2.3. Data Analysis

We used directed content analysis to analyze the data. This method is guided by theory or prior research and is a more structured process than traditional content analysis [34,35]. “The goal of a directed approach to content analysis is to validate or conceptually extend a theoretical framework or theory” [36] (p. 1281).

The first two authors began by reading through each transcript and identifying the key concepts for initial coding categories [35]. Next, based on stress and coping theory, which addresses how people cope with the adverse effects of stress [37], as well as prior research on moral distress theory and euthanasia [38], we determined operational definitions for each coding category. Next, we created an initial codebook as a guide for coding the remainder of the data. Then, a team of six graduate students, trained by the lead authors, utilized the code book to independently code the remaining interviews. Throughout the coding process, the students met with the lead authors to review new codes and to discuss discrepancies in the coding process. Data that could not be coded were noted and analyzed again later to assess whether they represented a new theme or a subcategory of an existing theme. One advantage of directed content analysis is that “existing theory can be supported and extended” [36] (p. 1281). Throughout the process, we kept meticulous notes of all code definitions, themes, and patterns within the data and tracked progress towards code and meaning saturation [39]. We continued to read interviews until we reached meaning saturation meaning that the interview data no longer elucidated nuanced meanings.

## 3. Results

The findings centered on seven key themes:

1. The need to be better prepared for crisis events;
2. Lack of public understanding;
3. Moral distress;
4. Empathy for others, especially young veterinarians;
5. Sources of support;
6. Pride, honor, and gratitude;
7. Emotional distancing and detachment—concerns external to one’s own mental health.

### 3.1. Better Preparation

All participants talked about the lack of preparation and the need to be better equipped to manage emergency public health livestock events. For instance, “I learned that we just need to be better prepared and in case it happens, at both the local and state level, all the way down, and so on. That’s what we’re working towards” (Participant E). In a similar vein, another veterinarian reflected upon the notion that even with planning, systems may not work effectively because of “the fragility of our system…” (Participant I), underscoring the fact that even with the best laid plans, events like the COVID-19 pandemic can create unforeseen circumstances that undermine preplanning.

### 3.2. Lack of Public Understanding

Many veterinarians voiced concern that the public is too far removed from food production to appreciate the need for depopulation. As one veterinarian expressed, “I tried to always explain what we were doing, you know, because some of our family is a little bit removed from agriculture. So, it’s a little harder to always get them to grasp the picture of why (we) would be doing that. And, you know, try to get everybody to understand that there are reasons it’s happening” (Participant A). Another veterinarian echoed frustration at the public’s misunderstanding of the complexity of the food chain process: “So, you know, I’d watch our local news and I think it wasn’t negative, it was, people didn’t really understand it, they didn’t understand why it was happening. I mean, the most negative thing you’d see would be like, ’We don’t understand why they don’t just give these things (animals] away?’ Or ’I don’t understand why they don’t just let them go.’ Or ’I would take them for free’" (Participant J).

### 3.3. Moral Distress

One involved participant aptly noted their moral distress—knowing the ethically correct action to take but feeling constrained from taking it—by stating, “Oh, absolutely…probably the most (moral distress) is when you’re actually in the act of doing it (depopulation) and you see a perfectly healthy pig” (Participant E). Another veterinarian added, “I didn’t doubt any of the decisions. I don’t. I’m not, you know, I was never upset or angry about, you know, this is the wrong decision. I think for me the hardest part was the one day I had to be present, or I was present for the ventilation shutdown…we can humanely euthanize pigs here today, or we cannot do it and they won’t have a home or market outlet. So, what choice did we have” (Participant H)?

Participants also talked about having to overcome judgment from people outside the industry: “I would say, yeah, some judgment, just in the fact that the industry was trying hard to accomplish an unbelievable task” (Participant A). Another participant talked about “being attacked” by the public and the media noting, “Yeah, they don’t understand a damn thing… I mean, it was like, at our most vulnerable, we were attacked. Um, you know, so you understand what military personnel feel like when, you know, they’re extremely vulnerable, and then they get attacked. And that was the case …. you just go man, this, it’s, you know, this isn’t what we want to be doing. It’s not what we’re in business to do. It’s what we had to do” (Participant L).

Similarly, another non-involved veterinarian observed “(Some people) didn’t want to come to work. They were sad and kind of aimlessly move through the day. Not excited about their jobs. All those types of things. And of course, what a horrible waste …when you consider how many people don’t have enough to eat around the world it is a horrible waste” (Participant C).

### 3.4. Empathy for Others

One participant noted, “And from my perspective, I have empathy for those young veterinarians who had to go through this process… And not just the veterinarians, also the farm workers, who also care about the pigs” (Participant D). In a related vein, another veterinarian observed: “I felt bad for the people who had to do it and who were involved in it, because that’s not something you ever want to do. They, like those farmers, they take great pride in their work and, and you’re producing those pigs for a purpose” (Participant B).

Another participant talked about the sympathy they felt for others: “So we understand that it’s emotional, for veterinarians, but more so for producers, because they put their heart and soul into raising their livestock and doing the best that they can and trying to make a living and trying to make a profit” (Participant C).

### 3.5. Sources of Support

Similar levels of community support were reported by both involved and not involved swine veterinarians. A veterinarian involved in depopulation talked about the supportive veterinary community: “I would say that I felt very supported (by swine veterinarians). I reached out to a couple of my colleagues and to run through the clinical situation and make sure that they agreed with my decision to euthanize. So, I felt very supported there and did not feel judged in that capacity” (Participant I).

In the view of another participant: “Just having a community around there and being able to talk openly about it helps a ton, even if it’s really a mental health service or not. But I mean, you have all these concerns and worries, and it’s there inside your head, they eat at you. But if you can talk it out with someone, they’ll be like, ‘Oh, I did that. And this is how it worked’ …that helps quite a bit” (Participant K). Finally, one swine veterinarian stated, “I would say that that organization was really good, really helpful, very supportive of the process” (Participant C).

### 3.6. Pride, Honor and Gratitude

One thing that stood out for the involved group of veterinarians were comments expressing pride, honor, gratitude, and being a hero. In their view, they were able to be there and oversee the process so that they could make the best of a horrific situation for the animals and the farmer. As one participant noted: “I don’t know, it’s kind of strange. I’m proud of it in a way. I mean, it was not a fun experience. It’s something I surely hope I don’t have to do again. But, I think we did a very good job with a completely new experience and a huge logistical challenge, a scientific challenge, and a welfare challenge. I’m proud that we managed the situation as well as we did. I’m proud that we did it in a way that we were essentially able to have veterinarians implement the program” (Participant J).

Another veterinarian noted the need to share experiences that can help promote animal welfare in these challenging situations: “We always do what’s right for the animal. And we share whatever, we have to make sure that what we learn in one place, if it works, we’ll share with other people because it’s what’s right for the animal” (Participant F).

### 3.7. Overarching Theme: Emotional Distancing and Detachment—Concerns External to One’s Own Mental Health

Interwoven throughout the swine veterinarians’ narratives were expressions of disbelief and shock at the devastating situation in which their profession found itself. Their training and professional experiences fell short in preparing them for the depopulation crisis. When responding to the industry’s action, veterinarians exclaimed “Oh, my goodness, can’t we figure this out” (Participant C)? They expressed incredulity that the profession was not “better prepared as an industry” and vexed, “How can we not solve this” (Participant K)? More seasoned veterinarians who had lived through several pandemics portrayed this mass depopulation as surpassing anything they had ever seen.

The veterinarians tended to *not speak* about their own feelings. This may be due to the vast sense of overwhelm and a potential emotional numbing: “Yeah, you intellectually understand it, but emotionally, you want to reject it as much as you can” (Participant G). Several veterinarians also alluded to the fact that swine veterinarians may not feel comfortable expressing their own feelings “because it’s a group of very high performing folks, very intelligent people who probably don’t want to always acknowledge that their struggling in life can be stressful, this career can be stressful” (Participant B). Since many of their family and friends were removed from their daily experiences in the depopulation process, the veterinarians felt isolated and withheld their feelings from those closest to them: “You know, talking about my feelings isn’t something I’m exactly good about anyway, and whining about my problems isn’t one either” (Participant I).

As with most traumatic experiences, those involved may experience an emotional distancing from the incident. When asked what they learned from the experience, one veterinarian proclaimed: “I wish I would have acknowledged earlier how stressful and traumatic the event was. I really repressed the feelings” (Participant L). A self-proclaimed “not super empathetic” veterinarian, posited that the high volume and task-centered nature of the work prohibited veterinarians from having “enough time to really think about the morality – maybe it’s just that (we’re] constantly moving and acting” (Participant D). They further stated, “There’s almost not a lot of time to kind of sit and think it’s really just like, ‘Okay, we’ve got to do this.’”

## 4. Discussion

The goal of this qualitative study was to better understand the experiences of U.S. swine veterinarians during the COVID-19-related depopulation efforts. The common themes that emerged from this study included: (1) the need to be better prepared for crisis events; (2) lack of public understanding; (3) moral distress; (4) empathy for others, especially young veterinarians; (5) sources of support; (6) pride, honor, and gratitude; and (7) the overarching theme of emotional distance and detachment—participants consistently expressed concerns external to their own mental health.

With respect to the overarching theme of emotional distance and detachment, we found that participants spoke often about their concerns related to others—farmers, producers, workers, and society. They spoke much less about their own emotional experiences. This was also reflected by the attention given in the interviews towards the suffering of the pig owners, farm workers, and others who had strong emotional and financial attachments to the swine. They recollected these experiences with empathy towards others while downplaying the emotional labor of their role in the depopulation. This type of response could possibly be a defense mechanism. It is possible that the participants were coping by distancing themselves, situating themselves within a system, and not speaking about their own feelings—except to express feelings of sympathy for others. Many times, when individuals experience an emotional or psychological shock, in an effort to self-protect, they respond by shutting off difficult emotions and feelings, making them difficult to access.

Another prevalent theme throughout the interviews was a sense of disbelief and distress that there was not a better solution to this type of problem. Many veterinarians expressed shock and dismay that the industry did not have a plan for this type of catastrophic event, including a wider array of options available for depopulation. Clearly, the field would benefit from allocating resources to researching alternative, welfare-minded options for depopulation when traditional methods are unavailable.

It is imperative that we use the knowledge gained from the COVID-19 pandemic- related depopulation experience to make improvements in the future. In addition to alternative depopulation methods, it is clear that further training related to depopulation is warranted. This training should include not only technical and logistical details, but ways to mitigate the potential negative psychological impact on all those involved.

This event also highlighted the need for additional, and ongoing, public education related to livestock production and reasons why depopulation is sometimes necessary. Public education campaigns to increase trust through enhanced transparency between the public and the livestock industry—including food animal veterinarians are suggested.

Lastly, the results of this study, along with numerous other studies examining the impact of depopulation efforts on those involved [9,10,11,38], speak to the need to prioritize mental health services for livestock/large animal veterinarians, with particular efforts aimed at helping to support veterinarians who may need to lead depopulation efforts. Part of this support should include acknowledging and recognizing the tendency of veterinarians to support their colleagues, as well as validating the feelings of pride and honor many feel for being able to respond quickly and efficiently to challenging situations that necessitate depopulation.

### Limitations

With respect to limitations, the participants were swine veterinarians who volunteered to be interviewed. It is possible that they are not representative of swine veterinarians in the United States. For example, the sample of individuals who participated in the quantitative survey who experienced higher levels of emotional distress may have been less likely to participate in a follow-up interview. Given the small number of swine veterinarians in the United States, we chose to not collect demographic information. We realized this would be a limitation, but we felt the need to ensure a maximum level of anonymity for this relatively small number of professionals sharing views on a potentially sensitive topic. In addition, the quantitative study occurred in the early months of the pandemic when levels of uncertainty and anxiety may have been higher than when we conducted the qualitative interviews which occurred at later stages in the pandemic when scientists better understood the mechanism of COVID-19 transmission. Understanding the retrospective nature of data collection, it is also possible that participants’ memories of these events may be skewed. Future research is needed to continue expanding our understanding of swine veterinarians’ mental health needs, not only for the next depopulation effort, but for everyday practice.

## 5. Conclusions

To minimize the negative emotional impact on veterinarians who perform mass depopulation, it is important to address the concerns of all those involved. It is important for the veterinary profession, food animal organizations, and academic scientists to all work together to develop educational opportunities and supportive services to veterinarians, others involved in depopulation efforts, and the public in a timely and coordinated manner.

It is also suggested that veterinary curricula include discussion about the behavioral health needs of veterinarians, veterinary support staff, and animal producers and farmers involved in mass depopulation events. This type of training could be incorporated into existing courses or continuing education opportunities [40]. Training for mental health experts on issues specific to mass depopulation, the human–animal bond, and the caring-killing paradox is also needed. It would be prudent for mental health experts to be well-versed in the psychosocial consequences of mass depopulation on participants. In summary, the depopulation event caused by COVID-19 caught the country by surprise. We have the opportunity to learn from this experience to create a better supportive structure for all those involved for the next inevitable pandemic.

## Data Availability

Data available upon request.
